# Inconsistencies over time in 5% of NetAffx probe-to-gene annotations

**DOI:** 10.1186/1471-2105-6-183

**Published:** 2005-07-20

**Authors:** Carolina Perez-Iratxeta, Miguel A Andrade

**Affiliations:** 1 Ontario Genomics Innovation Centre, Ottawa Health Research Institute, 501 Smyth Rd, Ottawa, Ontario K1H 8L6, Canada

## Abstract

**Background:**

DNA microarray probes are designed to match particular mRNA transcripts, often based on expressed sequences like ESTs, or cDNAs, many times incomplete. As a result, the relations between probes and genes can change as the sequence data are updated. However, it is frequent that the reported results of microarray analyses are given just as lists of genes without any reference to the underlying probes.

**Results:**

We show for a particular commercial microarray design that the number of probes associated to some genes change with time. These changes concern approximately 5% of the probe sets across the history of annotation releases over a two year span.

**Conclusion:**

We recommend to report probe set identifiers when publishing microarray results, and to submit those analyses to microarray public databases to ensure that the interpretation of the data is updated with the latest set of annotations.

## Background

During a large scale analysis of data derived using the Affymetrix MOE430 murine DNA microarray [1], we detected striking differences in the resulting set of expressed genes depending on whether we were using one or another release of the microarray probe annotations as distributed by Affymetrix (NetAffx [2]). This is due to probes that point to different genes in different versions of the NetAffx data (see for example, the assignment for probe set 1433436_s_at in Table 1). Considering that gene names are broadly used by researchers when reporting microarray analysis results and in order to assess the magnitude of these changes, we measured their kind and extent through the history of all annotation files using as example the Affymetrix MOE430A/B chips.

**Table 1:** Example of a pair of probe sets inconsistently annotated.

	1	2	3	4	5	6	7	8
probe set id	17-Mar-03	9-Apr-03	25-Jun-03	9-Oct-03	11-Dec-03	9-Apr-04	19-May-04	23-Jun-04
1433436_s_at	Thtpa	Thtpa	Thtpa	Ap1g2	Ap1g2	Thtpa	Thtpa	Thtpa
1419113_at	Ap1g2	Ap1g2	Ap1g2	Ap1g2	Ap1g2	Ap1g2	Ap1g2	Ap1g2

An example of a pair of probe sets inconsistently annotated across the history of the eight NetAffx annotation files. Probe sets 1433436_s_at and 1419113_at were both assigned to gene Ap1g2, the gamma subunit of adaptor protein complex AP-1 in versions 4 and 5. This is a Golgi apparatus gene involved in protein transport. Thtpa is a hydrolase enzyme, the thiamine triphosphatase. In our experimental data, 1433436_s_at was detected as present and 1419113_at as absent. NetAffx releases of October 9^th ^and December 11^th ^2003 would suggest that Ap1g2 was expressed, while any other release would give the opposite result.

Affymetrix DNA microarrays include probes for the detection of target sequences that are mainly based on UniGene clusters [3]. UniGene is a database of gene-oriented clusters of GenBank sequences, where in addition to sequences of well-characterized genes, hundreds of thousands of novel expressed sequence tag sequences (ESTs) have been included. Affymetrix probe sets are annotated according to their related current records in UniGene and LocusLink, including genomic location, gene symbol, and function description, when available (NetAffx database, [2]).

We obtained all 8 NetAffx releases for the MOE430A/B microarray, dated from 2003 March 17th until 2004 June 6th (kindly provided by Marco Raposo, Affymetrix). First, we observed that there was at least one gene name change for 13,699 of the approximately 45,000 probe sets included in the MOE430A/B chips. Many of these changes were simply probe sets initially without a gene name that were eventually associated to one. This reflects a general improvement in the functional annotation of the human genome. Other changes could be explained by the use of synonymous gene symbols. However, according to a table of synonymous gene symbols that we extracted from the LocusLink gene database [4], there was still a total of 2277 probe sets with gene name changes that could not be explained by the use of a synonym. This represents about the 5% of the total of probe sets in the chip.

The underlying problem is exemplified in Table 1, where it can be seen that at least one probe must have been temporarily assigned to the wrong transcript. These inconsistencies can be detected when two probe sets attached to the same gene in one version of the annotations are attached to different gene names in another version. Table 2 indicates the number of inconsistencies by pairs of probe sets observed from one version of NetAffx annotations to the next, which amounts to thousands. This explains the variation in the biological interpretation of an Affymetrix microarray experiment depending on the version of the NetAffx annotations used.

**Table 2:** Number of split and joined probe set pairs between consecutive versions of NetAffx.

**NetAffx versions**	**Splits**	**Joins**
1 → 2	0	0
2 → 3	5862	4140
3 → 4	2547	3575
4 → 5	1380	1742
5 → 6	5479	8787
6 → 7	0	0
7 → 8	4904	4553

Splits represent the number of pairs of probe sets that point to the same gene name in one NetAffx release but to a different gene name from each other in the following release. Joins represent the number of pairs of probe sets that point to different gene names from each other in a release but to the same gene name in the following release. For this computation, all probes with no gene name were considered as associated to a different gene name. Dates of the NetAffx versions are given in Table 1.

The design of DNA microarray probe sets is often based on assembled groups of expressed sequences observed as ESTs or cDNAs, and might represent partial transcripts. Additional evidence in the form of new sequences, or even new gene predictions, can modify the preliminary assignment (for example, by discovering that two ESTs that were considered to be representing different mRNA transcripts are actually part of the same one). Therefore, information assigned to a probe based on gene predictions (such as a gene name) can be considered non-static and might change over time. Although, one can expect annotations will improve over time due to more accurate genomic assemblies, the changes will still occur for a while since a large fraction of genes are still predicted. Probe sequences constitute the only static information attached to the microarray: this information is inherent to the design of the microarray and will not change over time. This was pointed out in the manuscript that describes the NetAffx annotation files [2] but currently there is no visible warning or reminder in the Affymetrix website.

It happens that, although these are implicitly well known facts in the bioinformatics community, experimental users of microarrays are not so aware of the problem, probably because the surprisingly large extent of these changes has not been pointed out before. For example, the recent letter from the Microarray Gene Expression Data Society [5] explains that deposition of microarray data in public databases assures data persistence, integration, accessibility, and data standardization, but misses the problem of variable gene structure. There are recent publications that deal with the analysis of relations between Affymetrix probe sets and gene sequences [6-8], but they do no report the extent of the variation of these relations along time as we have done here. This latter fact, which could convince many microarray users to send their data to public databases, has not been well publicized.

Deposition of microarray data in public databases is much more than just making the data public, but to making them really of use to the scientific community. Those databases include the descriptions of probe sequences and update constantly the non-static information associated to them, thus allowing the re-interpretation of the data and solving the problem we presented here.

## Authors' contributions

CP and MA participated in the design of the study, the computations, and preparation of the manuscript. Both authors read and approved the final manuscript.
